# Electrode montage-dependent intracranial variability in electric fields induced by cerebellar transcranial direct current stimulation

**DOI:** 10.1038/s41598-021-01755-9

**Published:** 2021-11-12

**Authors:** Jana Klaus, Dennis J. L. G. Schutter

**Affiliations:** Department of Experimental Psychology, Helmholtz Institute, Utrecht University, Heidelberglaan 1, 3584 CS Utrecht, The Netherlands

**Keywords:** Neuroscience, Computational neuroscience

## Abstract

Transcranial direct current stimulation (tDCS) is an increasingly popular tool to investigate the involvement of the cerebellum in a variety of brain functions and pathologies. However, heterogeneity and small effect sizes remain a common issue. One potential cause may be interindividual variability of the electric fields induced by tDCS. Here, we compared electric field distributions and directions between two conventionally used electrode montages (i.e., one placing the return electrode over the ipsilateral buccinator muscle and one placing the return electrode [25 and 35 cm^2^ surface area, respectively] over the contralateral supraorbital area; Experiment 1) and six alternative montages (electrode size: 9 cm^2^; Experiment 2) targeting the right posterior cerebellar hemisphere at 2 mA. Interindividual and montage differences in the achieved maximum field strength, focality, and direction of current flow were evaluated in 20 head models and the effects of individual differences in scalp–cortex distance were examined. Results showed that while maximum field strength was comparable for all montages, focality was substantially improved for the alternative montages over inferior occipital positions. Our findings suggest that compared to several conventional montages extracerebellar electric fields are significantly reduced by placing smaller electrodes in closer vicinity of the targeted area.

## Introduction

Transcranial direct current stimulation (tDCS) has become a commonly used tool to modulate spontaneous neuronal activity by polarizing superficial brain tissue through administering a weak continuous electric current through two or more electrodes affixed to the scalp^1,2^. In recent years, the cerebellum has gained increasing popularity as a target site to investigate motor and non-motor functions in healthy and clinical populations^3–10^. While proven effective in eliciting behavioural effects, absolute effect sizes are small to medium, and there is no evidence for polarity-dependent effects of anodal tDCS improving and cathodal tDCS worsening behavioural performance^10^. This suggests that cerebellar tDCS is subject to many factors potentially reducing its efficacy. One such factor may be the electrode montage used. With the traditionally low spatial resolution of tDCS, it is essential that the used electrode montage minimizes the surface area and intensity of the electric field elicited outside of the cerebellum.

In studies that aim to target a single cerebellar hemisphere, many researchers use either a buccinator or frontopolar montage^4,11^. For both montages, the active electrode is placed over the respective cerebellar hemisphere and the return electrode over the ipsilateral cheek (i.e., the buccinator muscle) or the contralateral supraorbital region, respectively. While proven effective in eliciting physiological^12^ and behavioural effects^10^, there have been no investigations into how focally these montages elicit an electric field in the targeted cerebellar hemisphere.

Another possible position for the return electrode is the ipsilateral deltoid muscle (i.e., the left or right shoulder). However, previous empirical work has demonstrated a linear decrease of the physiological effects of anodal tDCS with increasing distance between the active and the return electrode^13^, implying an overall lower electric field strength. Another study recently reported montage-independent effects of anodal tDCS on cerebellar brain inhibition, suggesting that there are no fundamental differences between buccinator, frontopolar, and deltoid montages^14^. In the current study, we therefore did not further evaluate the deltoid muscle as a position for the return electrode.

Computational modelling of electric field distributions provides a means to simulate the effects of current flow introduced by tDCS in the superficial parts of the brain that face the cranium. So far, there have been efforts to investigate the electric field distributions induced by electrode montages commonly used when targeting the cerebellum in healthy individuals^11,15–17^ and stroke patients^18,19^. However, while this work has convincingly shown that it is feasible to reach the cerebellar cortex with tDCS, several issues relating to focality, generalisability, and practicability remain. Most importantly, previous studies did not take into account electric fields induced outside of the target region, leaving open the possibility that the observed effects can, at least partly, be explained by additional modulation of extracerebellar regions. Furthermore, by limiting simulations to a small number of head models, previous studies did not address the interindividual variability of electric fields caused, for instance, by differences in anatomy. Of note, one study so far investigated differences in electric fields elicited in different age groups, but did not take individual variation within these groups into account^20^. Finally, some alternative montages require a complex setup of five instead of the conventionally used two electrodes^11^, potentially limiting their immediate applicability in typical research and clinical settings.

To address these issues, we performed a computational electric field modelling study targeting the right cerebellar hemisphere at 2 mA, using structural anatomical MRI scans of 20 healthy individuals. After examining field strengths and focality of the conventionally used buccinator and frontopolar montages, we explored six alternative montages aimed at limiting extracerebellar current spread while maintaining sufficiently high field strengths. For all montages, we assessed the elicited field strength and direction in the cerebellum as well as field focality as a proxy of how successful the respective montages are in affecting neuronal activity in the right posterior cerebellum only. Additionally, we measured the distance between the scalp and the cerebellar cortex for each individual and examined to which extent this measure is related to the field strength and focality of each montage.

## Experiment 1

### Methods

#### Participants

Twenty healthy individuals (eight female, mean age = 26.6 years, range: 21–38 years) were included from a publicly available dataset^21^. All participants were right-handed native German speakers with no known hearing or neurological impairment. Informed consent was obtained by all participants, and data acquisition was approved by the ethics committee of the Otto-von-Guericke University Magdeburg, Germany, and carried out in accordance with relevant regulations.

#### MRI scans

Both T1- and T2-weighted structural images were acquired on a 3.0 T Philips Achieva MRI scanner with a 32-channel head coil. T1-weighted images (274 sagittal slices; repetition time: 2500 ms; echo time: 5.7 ms; voxel size: 0.7 mm^3^; matrix: 384 × 384) were recorded with a 3D turbo field echo sequence (flip angle: 8°; SENSE reduction 1.2 along AP direction and 2.0 along RL direction). T2-weighted images (274 sagittal slices; repetition time: 2500 ms; echo time: 230 ms; voxel size: 0.7 mm^3^; matrix: 384 × 384) were recorded using a 3D turbo spin-echo sequence (TSE factor 105; bandwidth 744.8 Hz/px; SENSE reduction 2.0 along both AP and RL direction).

#### Electric field simulations

Simulations were performed with SimNIBS^22–24^. For each individual, tetrahedral head meshes were generated from T1- and T2-weighted structural MRI scans using the *headreco* function^25^. Electrode positions according to the International 10/10 EEG system were automatically calculated based on four fiducial points^26^.

**Figure 1:**
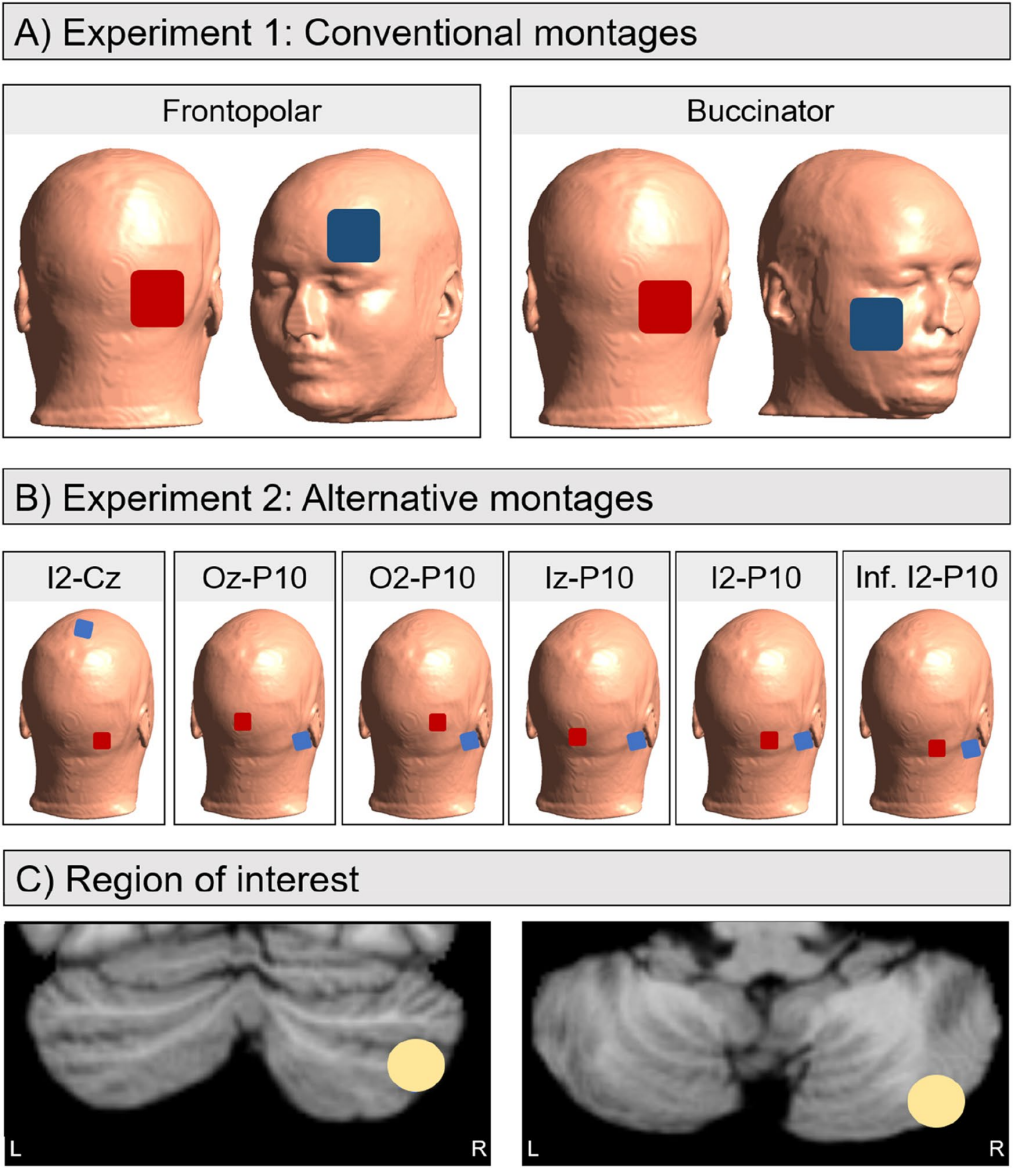
(**A** and **B**) Illustration of the electrode positions for conventional montages (**A**) and alternative montages (**B**). For the conventional montages, two electrode sizes were tested: 35 cm^2^ (not pictured) and 25 cm^2^. The alternative montages used 9 cm^2^ electrodes. Red electrodes refer to the anode, blue electrodes refer to the cathode. (**C**) Illustration of the region of interest (MNI coordinates: x = 40, y = − 76, z = − 46; 5 mm radius) used in the current study, plotted on coronal (top) and axial (bottom) views of a standard MNI brain.

The frontopolar and buccinator montages were simulated for each individual (Fig. 1A). For both electrode montages, the anode was placed over I2. For the buccinator montage, the cathode was placed over the right cheek and for the frontopolar montage over the left orbit (electrode position Fp1). Both montages were tested with 35 cm^2^ and 25 cm^2^ rectangular electrodes.

Except for the varying electrode size, identical simulation parameters were used for all montages. Electrodes of 2 mm thickness were simulated to be contained within a 5 mm thick sponge whose size extended the electrode size by 0.5 mm on both edges (e.g., for a 5 × 7 cm electrode we simulated a sponge of 5.5 × 7.5 cm). Stimulation intensities were set to −2 mA at the anode and 2 mA at the cathode. Standard conductivity values were used (σ_WM_ = 0.126 S/m, σ_GM_ = 0.275 S/m, σ_CSF_ = 1.654 S/m, σ_scalp_ = 0.465 S/m, σ_skull_ = 0.01 S/m, σ_eye balls_ = 0.500 S/m, σ_electrode rubber_ = 29.4 S/m, σ_sponge/gel_ = 1.0 S/m)^27^.

For each simulation and individual, field focality values were extracted from the fields summary output generated by SimNIBS. Specifically, we used the values corresponding to the area of the grey matter with field strengths equal to or higher than 75% of the 99.9th percentile. Higher values represent larger spread and thus lower focality. Field directions were extracted from the simulations using custom Python scripts.

#### Region of interest

The region of interest (ROI) was located in the right posterior cerebellum, corresponding to MNI coordinates *x* = 40, *y* = − 76, *z* = − 46, with a radius of 5 mm (Fig. 1C). This matches lateral regions of right Crus II, just inferior to the horizontal fissure. Using Python (version 3.7.10), the ROI was transformed to individual subject space using the *mni2subject_coords* function in SimNIBS, and subsequently the average field strength for this region was extracted, with higher values corresponding to stronger electric fields.

#### Scalp–cortex distance

To determine the potential influence of the distance between the scalp and the cerebellar cortex of the target region, manual measurements were performed for each individual. The T1-weighted structural scans were converted to DICOM files using the N2D programme (version 1.11.0, http://dicomapps.com). Manual measurements were performed independently both in the axial (DS) and the sagittal (JK) view in MicroDicom (version 3.8.1, http://www.microdicom.com). Distance from the scalp to the cerebellar cortex was measured as the shortest perpendicular connection between the outer surface of the scalp and five random locations along the horizontal fissure separating right Crus I and II. Axial and sagittal measurements were highly correlated (*r*_18_ = 0.937, *p* < 0.0001) and averaged for further analyses.

#### Statistical analyses

All statistical analyses were computed in R (version 3.6.0). Normality of the dependent variables per montage was confirmed with Shapiro–Wilk tests using the *shapiro.test* function (all *W*s > 0.91, all *p*s > 0.075). To compare both mean field strengths and field focality across montages, Bonferroni-corrected pairwise *t* tests, in which the *p* values are multiplied by the number of comparisons, were calculated using the *pairwise.t.test* function. Pearson’s correlations between both field strength and focality and individual scalp–cortex distance were calculated using the *cor.test* function. For all analyses, the *α* level of significance was set to < 0.05 (two-tailed, Bonferroni-corrected). Figures were created using a combination of *ggplot2* (version 3.3.2^28^) and *ggpubr* (version 0.4.0^29^).

### Results

Table 1 reports descriptive statistics for the average field strength in the right posterior cerebellum as well as field focality for the four investigated montages.

**Table 1:** Mean field strength at the ROI and field focality for each of the four tested conventional electrode montages.

	Buccinator 35 cm^2^	Buccinator 25 cm^2^	Frontopolar 35 cm^2^	Frontopolar 25 cm^2^
**Mean field strength at ROI (V/m)**				
Mean (*SD*)	0.325 (0.112)	0.358 (0.129)	0.386 (0.131)	0.415 (0.146)
Median [Min, Max]	0.306 [0.150, 0.599]	0.332 [0.158, 0.654]	0.363 [0.175, 0.685]	0.386 [0.181, 0.722]
**Field focality (cm**^**3**^**)**				
Mean (*SD*)	11.3 (4.3)	9.3 (3.7)	10.5 (3.6)	10.4 (3.3)
Median [Min, Max]	10.0 [5.5, 19.2]	9.0 [3.8, 15.1]	10.3 [5.4, 17.9]	9.6 [5.6, 18.8]

#### Average field strength in right posterior cerebellum

**Figure 2:**
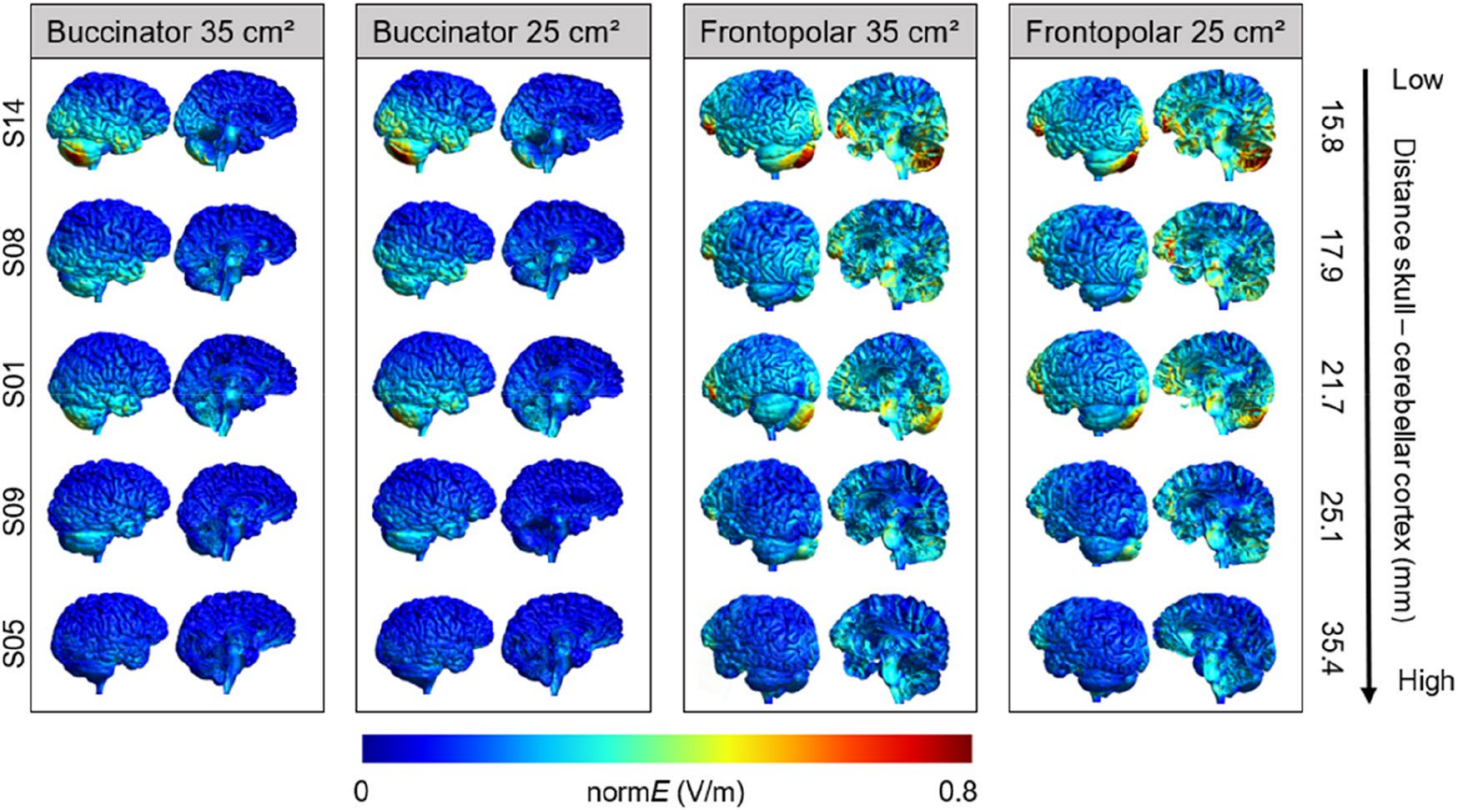
Field strength (normE in V/m) for the four conventional electrode montages. Individual electric fields are depicted for five individuals representative of the range of scalp–cortex distances. An illustration of individual electric fields of all individuals included in the study can be found in the “Supplementary Materials” (Fig. S1).

The highest field strength at the ROI was obtained with the frontopolar montage using 25 cm^2^ electrodes. However, none of the comparisons across montages were statistically significant (all *p*s > 0.190), indicating that there were no reliable differences in the electric field induced at the ROI across montages at the group level. In contrast, field strengths injected by the respective montages differed substantially across individuals (see Fig. 2 for five representative individuals), with individual averages ranging from 0.150 to 0.722 V/m (see Table 1).

#### Field focality

Whereas field focality did not differ between the four montages (all *p*s > 0.550), there were considerable differences across montages in the extracerebellar spread of the electric field both on the individual and the group level (Fig. 2). The buccinator montages induced electric fields of up to 0.3 V/m in the right inferior and anterior portions of the temporal lobe, right inferior occipital regions, and brainstem. Additionally, lower electric fields (~ 0.1–0.2 V/m) were observed throughout large parts of the right hemisphere. Placing the cathode over the left supraorbital region caused stronger electric fields (0.3–0.5 V/m) in the left prefrontal, anterior temporal, and right inferior occipital cortex, as well as lower electric fields (~ 0.1–0.2 V/m) throughout the entire cerebral cortex. Notably, substantial electric field distributions were also observed in the brainstem, corpus callosum, and cingulate cortex. Here, we limited our analysis of field focality to the area of the grey matter with field strengths equal to or higher than 75% of the 99.9th percentile. However, estimations of electric field focality at a threshold of 50% yielded comparable results, demonstrating conceptual threshold-independence of our findings (see “Supplementary Materials”, Fig. S5).

#### Influence of scalp–cortex distance

**Figure 3:**
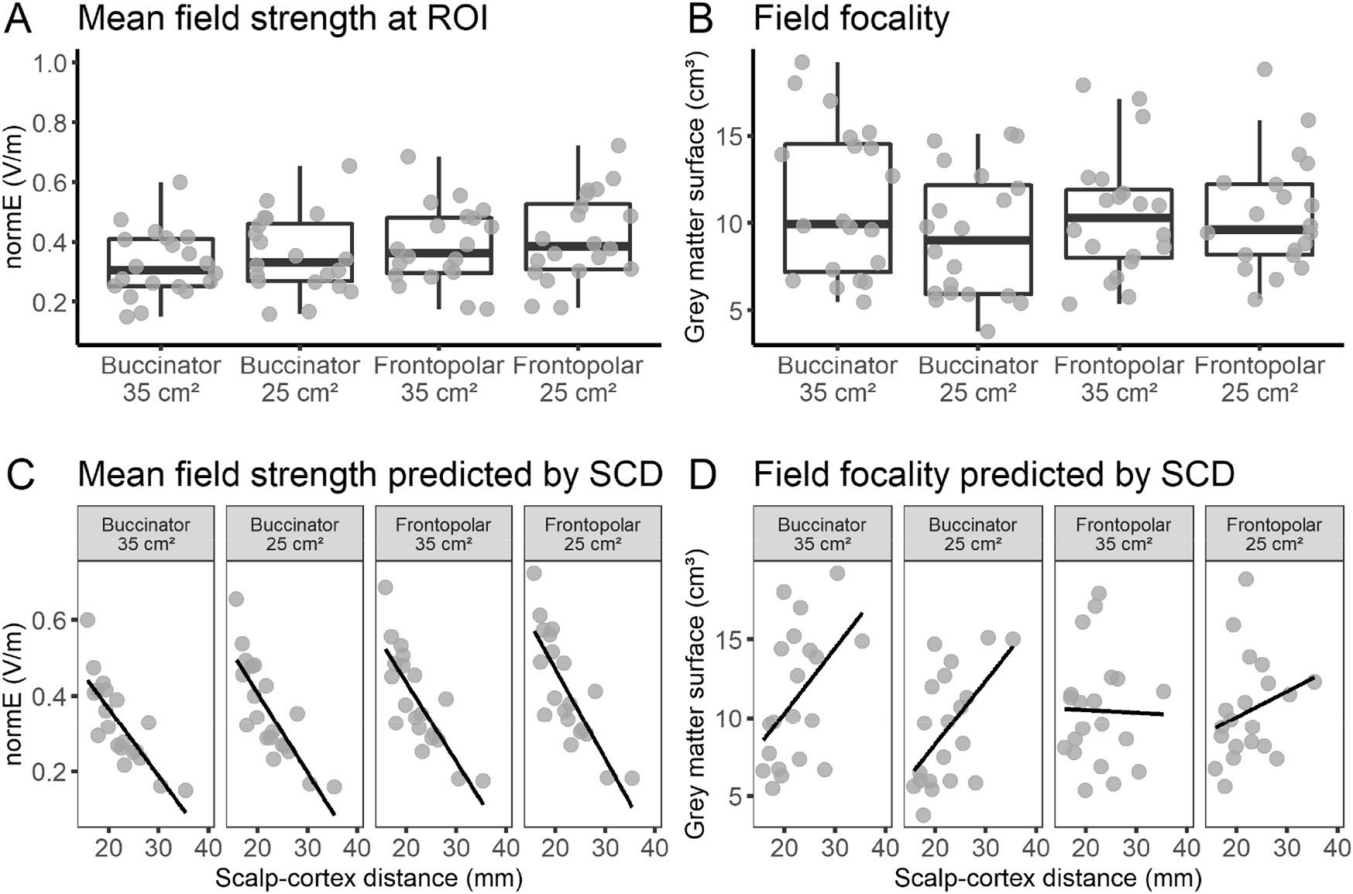
Overview of descriptive values of (**A**) mean field strength and (**B**) field focality for the four conventional montages tested in Experiment 1, as well as their association with individual scalp–cortex distance (SCD; **C**, **D**). Individual points correspond to individual head meshes.

The distance between the scalp and the right cerebellum varied substantially between individuals (mean = 22.3 mm, *SD* = 4.9, range: 15.8–35.4). Scalp–cortex distance and mean field strength at the ROI were negatively correlated for all montages (all *r*s < −0.814, *p*s < 0.001; Fig. 3C), suggesting that independent of electrode positions, the magnitude of the field strength linearly decreased with increasing distance between electrodes and the cerebellar surface. By contrast, field focality and scalp–cortex distance were only correlated for the buccinator montages (35 cm^2^: *r* = 0.481, *p* = 0.032; 25 cm^2^: *r* = 0.556, *p* = 0.011), but not for the frontopolar montages (35 cm^2^: *r* = -0.023, *p* = 0.923; 25 cm^2^: *r* = 0.253, *p* = 0.282; Fig. 3D).

#### Direction of current flow (normal component)

**Figure 4:**
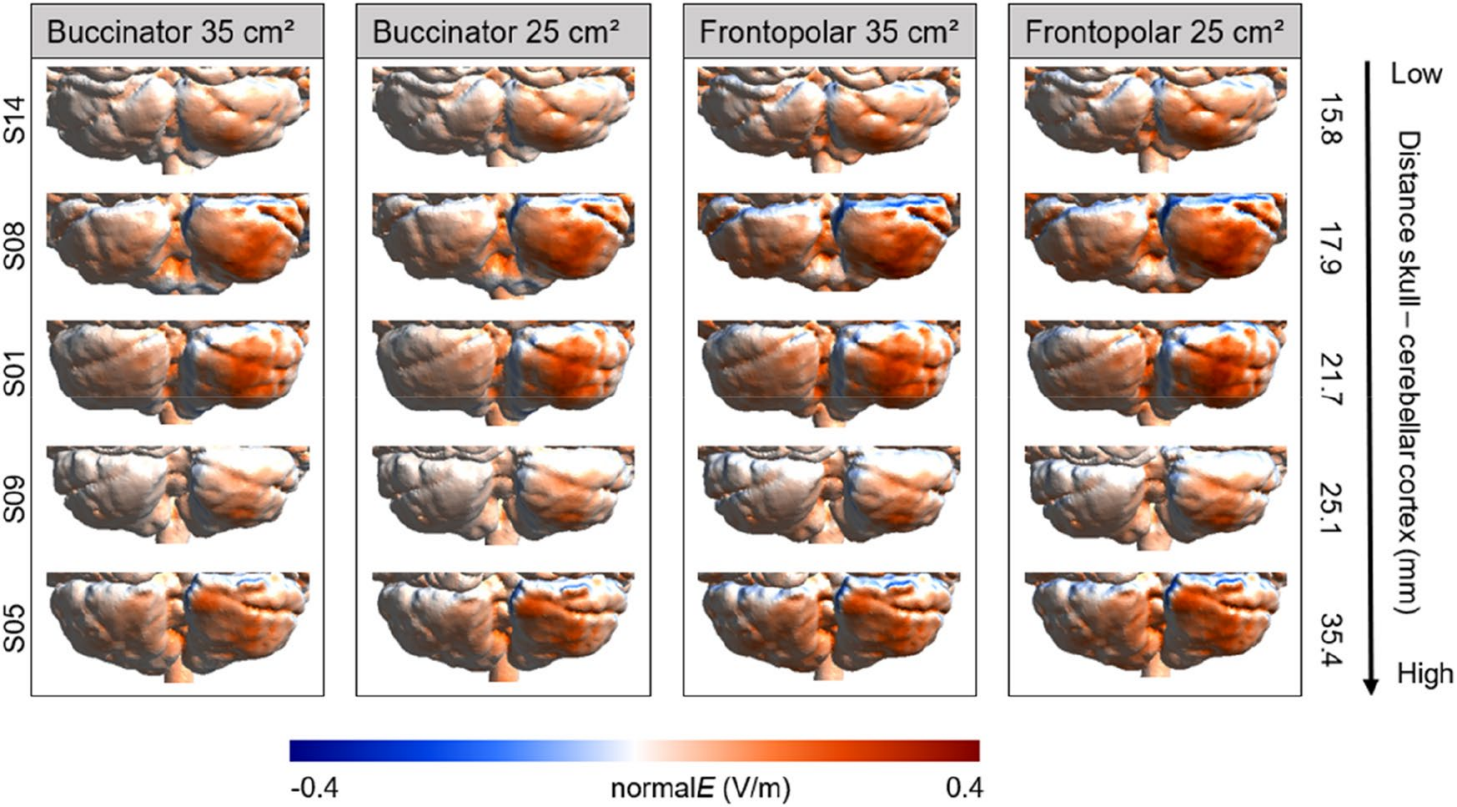
Field direction (normalE in V/m) for the four conventional electrode montages. Individual electric fields are depicted for five individuals representative of the range of scalp–cortex distance. Positive values (red) refer to current inflow and negative values (blue) refer to current outflow. An illustration of individual field directions of all individuals included in the study can be found in the “Supplementary Materials” (Fig. S2).

As illustrated in Fig. 4, all montages resulted in a predominantly inflowing current field in the right cerebellar hemisphere, owing to the fact that the anode was placed over this location. The position of the cathode (Fp1 for the frontopolar montage and the right buccinator for the buccinator montage) did not affect this pattern.

### Interim summary

Simulating electric field distributions for two conventionally used electrode montages and two different electrode sizes to target the right posterior cerebellum provided similar values of mean field strength and focality. Average field strengths within the region of interest at a stimulation intensity of 2 mA were comparable to previous cerebellar modelling studies^11,14,17^. Moreover, scalp–cortex distance was negatively correlated with the average field strength for all montages, which is in line with previous work on tDCS of non-cerebellar regions^27,30^. Importantly, while all montages resulted in current inflow at the ROI, none of them exclusively targeted the right cerebellar hemisphere, as indicated by the simulations showing relatively large electric fields with substantial spread outside of the target region. Additionally, an association of scalp–cortex distance and field focality was only found for the buccinator montages. This implies that the relationship between scalp–cortex distance and field focality is diminished with increasing distance between the electrodes, as is the case for the frontopolar montage.

While conventionally used electrode montages on average do seem to target the cerebellar hemisphere, particularly their focality can be improved. In Experiment 2, we therefore tested six alternative montages which attempted to tackle this issue on several levels. First, the surface area of the electrodes was reduced to 9 cm^2^, as previous work has shown that smaller electrodes substantially improve focality of the injected electric field^31–33^. Furthermore, we investigated various electrode position configurations which systematically decreased the distance to the target region. Based on the findings from the first experiment, we reasoned that decreasing the distance between the anode and the cathode would reduce the size of the electric field, allowing for a more focal distribution.

## Experiment 2

### Methods

Head reconstructions were used from the same participant sample as in Experiment 1, and electric field distributions were examined in the same region of interest in the right posterior cerebellum (Fig. 1C).

#### Electric field simulations

Six electrode positions in which the position of both electrodes was systematically moved closer to the target region were investigated (Fig. 1B). In particular, different setups involving EEG positions I2, Iz, Cz, Oz, O2 and P10, as well as one montage placing both electrodes approximately 2 cm below I2 and P10 (henceforth inferior I2–P10) were investigated. The rationale behind these electrode configurations was that, as shown in Experiment 1, field focality is improved by decreasing the distance between the two electrodes, and we therefore investigated different setups centred around the right cerebellar hemisphere. Furthermore, to increase current density of the electrodes, thus creating a stronger, less diffuse electric field, the electrode size was reduced to 3 × 3 cm, with sponges of size 3.5 × 3.5 cm. Except for these changes, the electric field simulations were performed with the same parameters and following the same procedure as in Experiment 1.

#### Statistical analyses

Average field strength values were normally distributed (all *W*s > 0.93, all *p*s > 0.176), and differences between mean field strengths across montages were thus evaluated with pairwise *t* tests. For field focality, Shapiro–Wilk tests indicated deviation from the normal distribution for four of the six montages (I2–Cz: *W* = 0.88, *p* = 0.017; Oz–P10: *W* = 0.88, *p* = 0.017; I2–P10: *W* = 0.77, *p* < 0.001; inferior I2–P10: *W* = 0.87, *p* = 0.013; O2–P10: *W* = 0.94, *p* = 0.290; Iz–P10: *W* = 0.92, *p* = 0.118). We therefore computed pairwise Wilcoxon rank sum tests using the *pairwise.wilcox.test* function with Bonferroni correction for multiple comparisons to evaluate field focality differences between montages. Associations between scalp–cortex distance and field strength were investigated with Pearson’s correlations, and associations between scalp–cortex distance and field focality with Spearman correlations. For all analyses, the α level of significance was set to < 0.05 (two-tailed, Bonferroni-corrected).

### Results

Table 2 reports descriptive statistics for average field strength in the right posterior cerebellum as well as field focality for the six investigated montages.

**Table 2:** Mean field strength at the ROI and field focality for each of the six tested alternative electrode montages.

	I2–Cz	Oz–P10	O2–P10	Iz–P10	I2–P10	Inferior I2–P10
**Mean field strength at ROI (V/m)**						
Mean (*SD*)	0.473 (0.156)	0.487 (0.204)	0.449 (0.194)	0.453 (0.192)	0.421 (0.186)	0.353 (0.161)
Median [Min, Max]	0.449 [0.215, 0.793]	0.437 [0.205, 0.898]	0.397 [0.184, 0.848]	0.402 [0.177, 0.848]	0.379 [0.156, 0.816]	0.321 [0.131, 0.686]
**Field focality (cm**^**3**^**)**						
Mean (*SD*)	12.6 (5.9)	7.5 (2.1)	4.8 (1.4)	8.8 (2.7)	5.7 (2.3)	4.2 (1.2)
Median [Min, Max]	9.7 [5.2, 24.9]	7.0 [3.8, 12.4]	4.9 [2.8, 7.7]	8.8 [5.1, 13.7]	5.10 [3.4, 13.7]	3.8 [2.8, 7.1]

#### Average field strength in right posterior cerebellum

**Figure 5:**
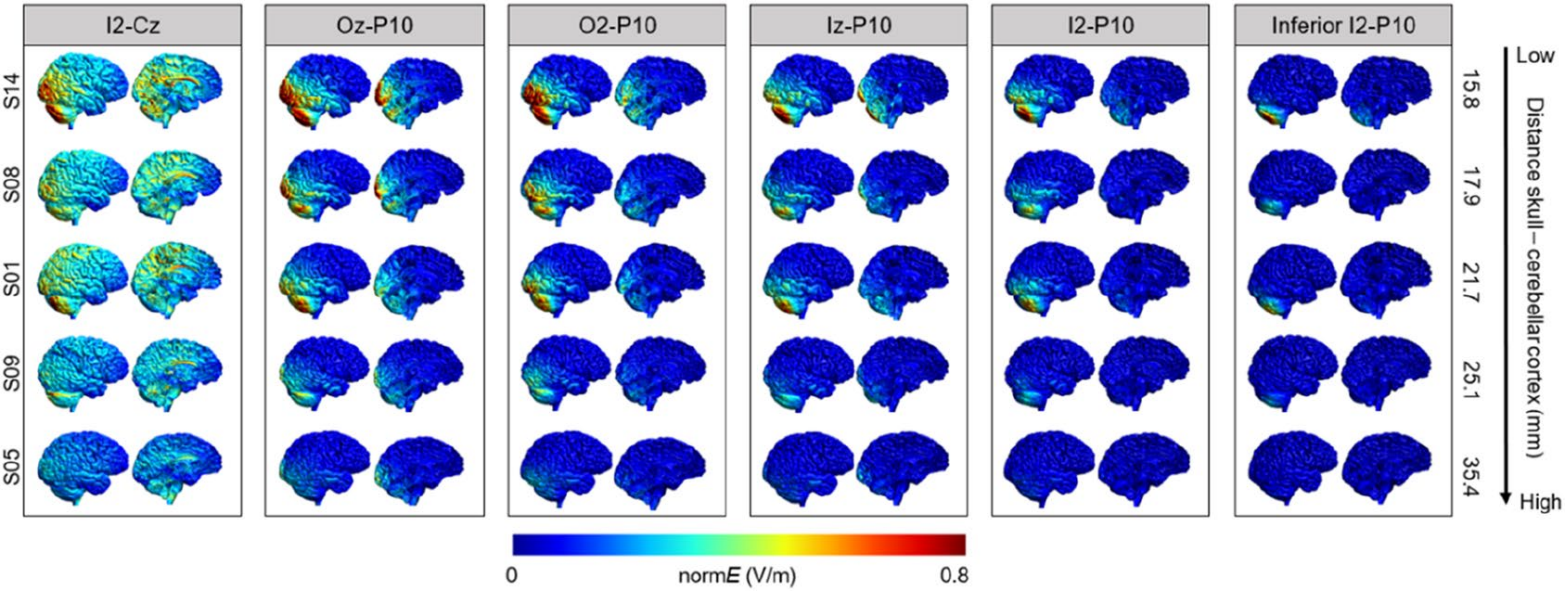
Field strength (normE in V/m) for the six alternative electrode montages. Individual electric fields are depicted for five individuals representative of the range of scalp–cortex distance. An illustration of individual electric fields of all individuals included in the study can be found in the “Supplementary Materials” (Fig. S3).

**Figure 6:**
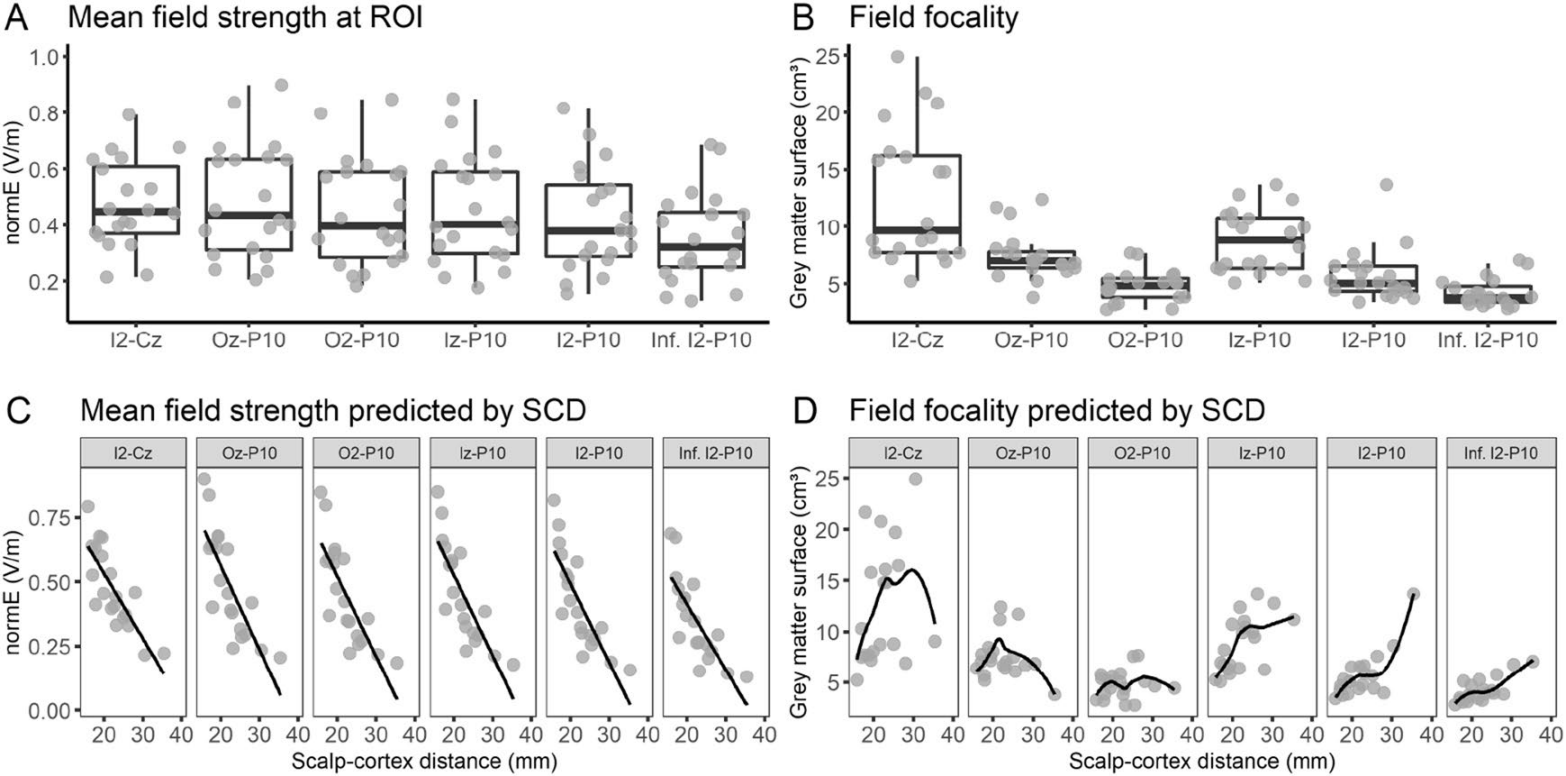
Overview of descriptive values of (**A**) mean field strength and (**B**) field focality for the six alternative montages tested in Experiment 2, as well as their association with individual scalp–cortex distance (SCD; **C**, **D**). Individual points correspond to individual head meshes.

Figure 5 illustrates modelled electric fields for five representative subjects. Mean field strengths in the region of interest did not differ across the six montages (*p*s < 0.340; Fig. 6A). As for the conventional montages, there was considerable interindividual variability, with field strength averages as low as 0.131 V/m and as high as 0.898 V/m (Table 2). No differences in mean field strength between the conventional and alternative montages were observed (*p*s > 0.092).

#### Field focality

As illustrated in Fig. 5, the alternative montages affected field focality to different extents. Extracerebellar current spread was lowest with the I2–P10 and inferior I2–P10 montages. There were no significant differences in field focality between Oz–P10 and Iz–P10, O2–P10 and I2–P10, and O2–P10 and inferior I2–P10 (*p*s > 0.999). All other montages significantly differed from each other (*p*s < 0.047), with O2–P10 and inferior I2–P10 exhibiting the best focality (Fig. 6B). Compared to the conventional montages, only the O2–P10 (*p*s < 0.005), I2–P10 (*p*s < 0.005), and inferior I2–P10 montage (*p*s < 0.0001) showed consistently improved field focality. Note that as for Experiment 1, estimations of electric field focality at a threshold of 50% instead of 75% yielded comparable results (see “Supplementary Materials”, Fig. S5).

#### Influence of scalp–cortex distance

Scalp–cortex distance and mean field strength at the ROI were negatively correlated for all montages (all *r*s < −0.794, all *p*s < 0.0001; Fig. 6C). As for the conventional montages, the magnitude of the field strength linearly decreased with increasing distance between the electrodes and the cerebellar cortex. Furthermore, field focality and scalp–cortex distance were positively correlated for the three montages which placed both electrodes below occipital electrode positions (Iz–P10: *rho* = 0.664, *p* < 0.002; I2–P10: *rho* = 0.625, *p* < 0.004; inferior I2–P10: *rho* = 0.693, *p* < 0.001; Fig. 6D), indicating that lower scalp–cortex distance was associated with more focal electric fields for these montages. No such association was found for the other three montages (I2–Cz: *rho* = 0.395, *p* = 0.085; Oz–P10: *rho* = − 0.077, *p* = 0.748; O2–P10: *rho* = 0.014, *p* = 0.955).

#### Direction of current flow (normal component)

**Figure 7:**
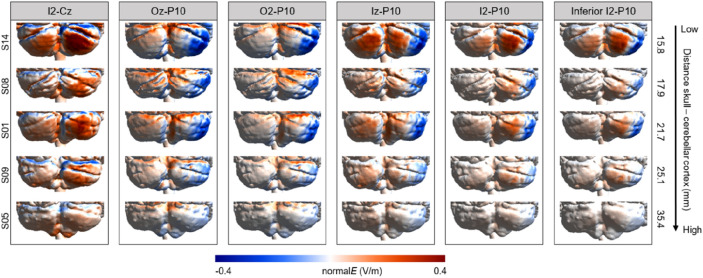
Field direction (normalE in V/m) for the six alternative electrode montages. Individual electric fields are depicted for five individuals representative of the range of scalp–cortex distance. Positive values (red) refer to current inflow and negative values (blue) refer to current outflow. An illustration of individual field directions of all individuals included in the study can be found in the “Supplementary Materials” (Fig. S4).

As illustrated in Fig. 7, decreasing the distance between the electrodes resulted in a more pronounced dissociation between inflow-outflow currents in the right cerebellar hemisphere. While the I2–Cz montage, for which the electrodes are spaced relatively far away from each other, was more comparable to the conventional montages in terms of current field direction, the other alternative montages resulted in current inflow closer to the medial part of the cerebellum and current outflow in the right lateral regions.

## General discussion

This study examined the electric field distributions of conventional and alternative two-electrode montages for targeting the right posterior cerebellum with tDCS. Results show that while all montages elicited a comparably strong electric field in the right posterior cerebellum, field focality was considerably improved in the I2–P10 and inferior I2–P10 montages where the electrodes were placed in close proximity over the right cerebellar hemisphere. Moreover, simulation findings of the field directions indicated predominantly current inflow in the right cerebellum for the conventional montages, and current inflow around the medial cerebellum and current outflow at the lateral right cerebellar hemisphere for the alternative montages in which the electrodes were placed in closer vicinity to each other. All the montages showed large interindividual variability in the electric field distributions. These differences across individuals could be an important contributor to the variability in reported cerebellar tDCS effects observed across studies, requiring recruitment of relatively large participant samples to reach sufficient statistical power.

### Montage-specific measures of field strength and focality

Across all montages, comparable average field strengths of ~0.35 V/m (range: 0.31–0.41) were observed over the right posterior cerebellum. This shows that, on the level of the ROI, all montages were successful in targeting the posterior cerebellar region at the proposed minimal mean field strength needed to polarize neural tissue^1,34^.

In line with the empirical fact that electric fields become more focal with decreasing physical distance between electrodes^31–33^, focality of the alternative montages with tightly spaced electrodes over the right cerebellar hemisphere was higher for both the conventional montages and those alternative montages which placed one electrode away from the cerebellar hemisphere. In fact, field focality was almost twice as high in the (inferior) I2–P10 montages as compared to the conventional and some of the alternative montages (i.e., I2–Cz, Oz–P10, and Iz–P10; see Tables 1 and 2). Interestingly, numerically, the O2–P10 montage was not distinguishable from the (inferior) I2–P10 montages. However, when taking a closer look at the actual electric fields (Fig. 5 and Supplementary Fig. S3), it becomes apparent that these favourable numbers do not imply that this montage was as successful in focally targeting the ROI. In fact, field focality as reported in this paper merely quantifies how focal the electric field is *anywhere* in the brain, not in the ROI itself. Therefore, the O2–P10 montage elicited a focal electric field superior to the target region, including the right occipital cortex.

The electric field distribution of the conventional montages suggests that previous studies using these electrode montages may have stimulated additional extracerebellar regions. For the buccinator montage, additional electric fields were observed primarily in the ipsilateral inferior temporal lobe. The frontopolar montages caused electric fields larger than 0.1 V/m (range: 0.07–0.50) in essentially the entire cerebral cortex, with peaks in the contralateral prefrontal and ipsilateral occipital cortex (Fig. 2). This suggests that particularly the frontopolar montage is less suited to exclusively target the cerebellum.

It is important to note that while the current study demonstrates improved focality of the (inferior) I2–P10 compared to the conventional and the other alternative montages, it remains an open question whether these alternative montages will indeed yield less variable behavioural and neurophysiological effects. Future research could examine whether reducing extracerebellar spread will result in more homogeneous results across individuals and studies.

### Individual differences in elicited field strength

Simulating electric fields in 20 different head meshes allowed us to study interindividual variability in achieved field strength, direction and focality (see Figs. 2, 4, 5, and 7, and Supplementary Figs. S1–S4). Across all investigated montages, on average 68% of individuals had an average field strength larger than 0.3 V/m in the right cerebellum (frontopolar 35 cm^2^: 70%; frontopolar 25 cm^2^: 80%; buccinator 35 and 25 cm^2^: 55% each; I2–Cz: 90%; Oz–P10: 80%; O2–P10 and Iz–P10: 70% each; I2–P10: 60%; inferior I2–P10: 50%). These percentages of potential responders are similar to the findings reported in a previous study in which tDCS was applied to the motor cortex^35^. Individual differences in anatomy, including skull thickness and cerebrospinal fluid volume, likely influence the efficacy of tDCS to induce effects on cerebellar physiology^27,36^. Here, we demonstrate that the magnitude of the elicited electric field in the ROI decreases with increasing distance between the targeted grey matter and the scalp. Furthermore, field focality improved with lower scalp–cortex distance for the buccinator and alternative montages which placed both electrodes below occipital electrode positions. In sum, assessing individual scalp–cortex distance based on structural MRI scans may help to reduce variability and even allow for titrating current intensity in future studies.

Finally, a limitation of the study is that we employed an intuitive approach instead of automated optimization tools to examine alternative electrode configurations by systematically decreasing the distance between the two electrodes, centered around the right cerebellar hemisphere. However, the results from Experiment 2 indicate that focality increases with decreasing distance between the electrodes, suggesting that the alternative montages (specifically I2–P10 and inferior I2–P10) provide a valid approximation of the target focality of the improved electrode montages. Crucially, we acknowledge that if researchers can in fact obtain individual MRI scans for their participants, subject-specific optimization procedures can and should be employed to determine electrode montages adapted to individual anatomic differences to achieve the best focality^11,20^. Such an approach would allow for an individually adjusted electrode montage which would further reduce the between-participant variability when targeting the cerebellum with tDCS.

## Summary and conclusion

With the present electric field simulations aimed at targeting the right posterior cerebellum, we have shown that conventional electrode montages are suboptimal in eliciting focal electric fields in the cerebellum. Two alternative montages using smaller electrodes at closer proximity demonstrated improved field focality while maintaining comparable field strengths and substantially reduced extracerebellar current spread, while four other alternative montages were less successful. Further, we show that while interindividual variability in field strengths is strongly associated with scalp–cortex distance for all montages, the relationship between field focality and scalp–cortex distance is strongest for the montages placing both electrodes below occipital electrode positions. This provides new avenues for future research aiming at reducing heterogeneity in cerebellar tDCS studies by creating a more focal electric field at posterior sites of the cerebellum. We demonstrate that this can be achieved with a simple setup of two smaller electrodes, making these montages immediately applicable with conventional tDCS setups. Researchers may preferably use the small electrodes so as to allow for a sufficient inter-electrode distance, thus avoiding excessive shunting of the current across the scalp. Given the better focality of the inferior I2–P10 compared to I2–P10 montage, we advise researchers to place the centre of the electrodes approximately 2 cm below the electrode positions I2 and P10.

## Supplementary Information

Supplementary Figures.

## Data Availability

The data associated with this manuscript are available on the Open Science Framework (https://osf.io/yh6j8/?view_only=0f300f77a370473f8c7b1cdaa858d7ee). Note that this repository is currently private. It will be made publicly available upon publication of this manuscript.
