# Supplementary material for: Electrode montage-dependent intracranial variability in electric fields induced by cerebellar transcranial direct current stimulation
Source: Sci Rep. 2021 Nov 12;11:22183. doi: 10.1038/s41598-021-01755-9 (PMC8589967; doi:10.1038/s41598-021-01755-9)

Figure S 1. Electric field distributions for the four conventional montages tested in Experiment 1.

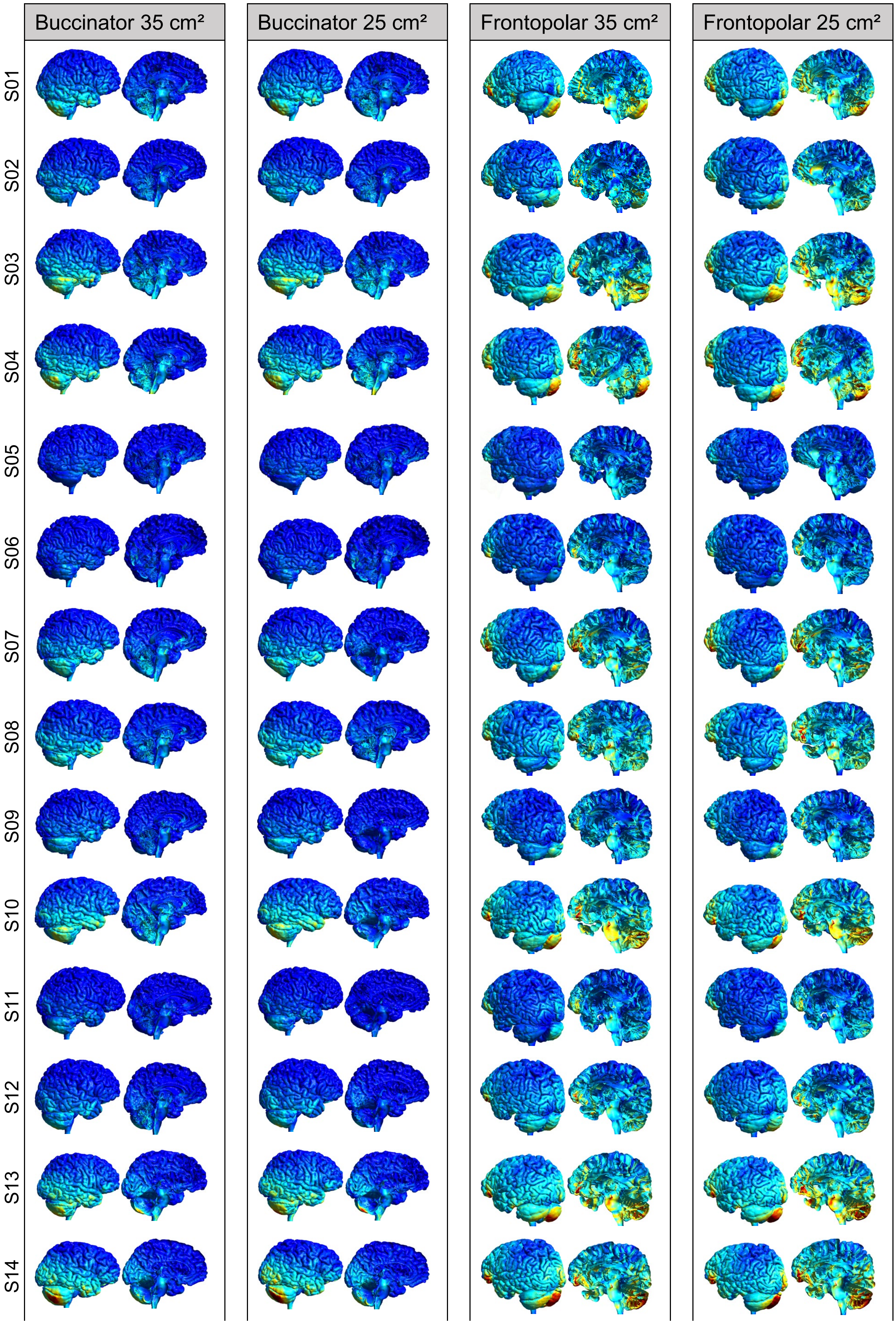

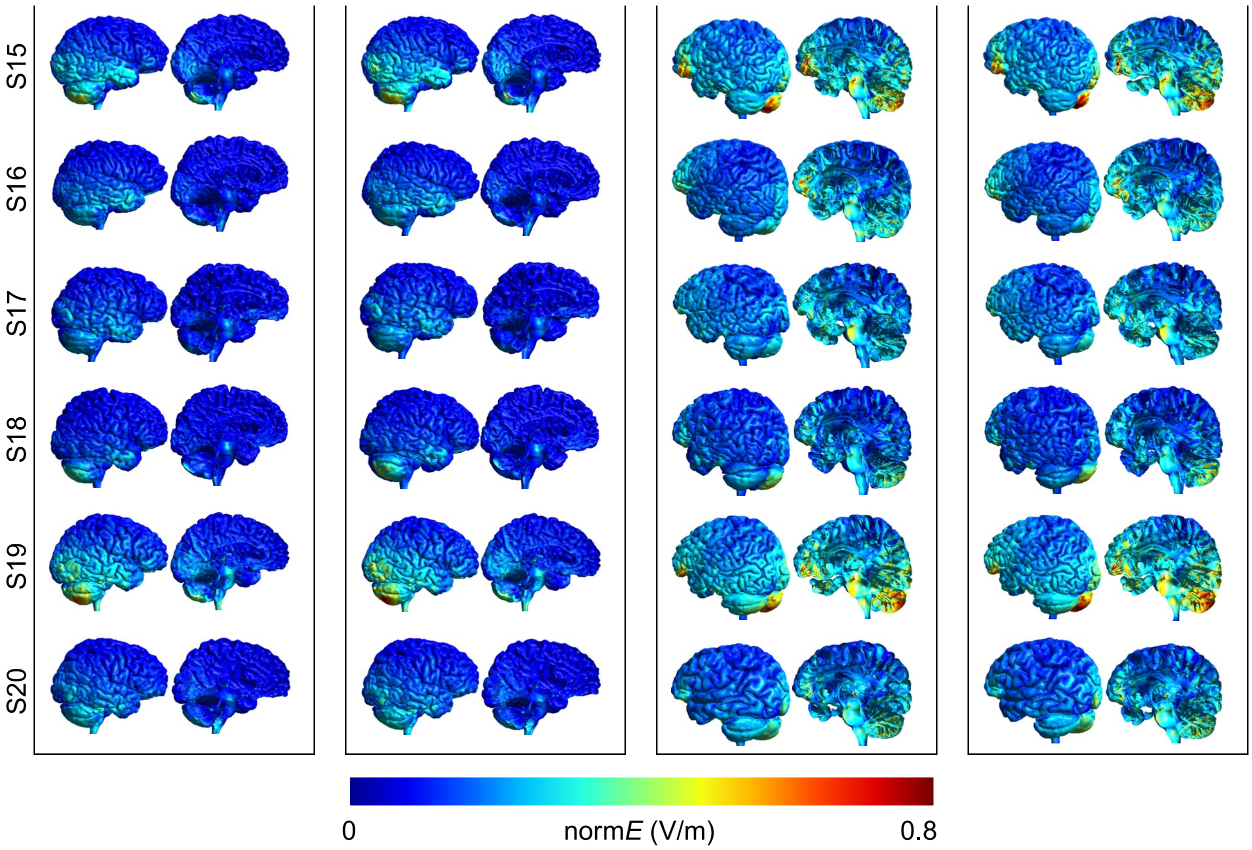
S15
S16
S17
S18
S19
S20
0
normE (V/m)
0.8

Figure S 2. Electric field directions for the four conventional montages tested in Experiment 1.

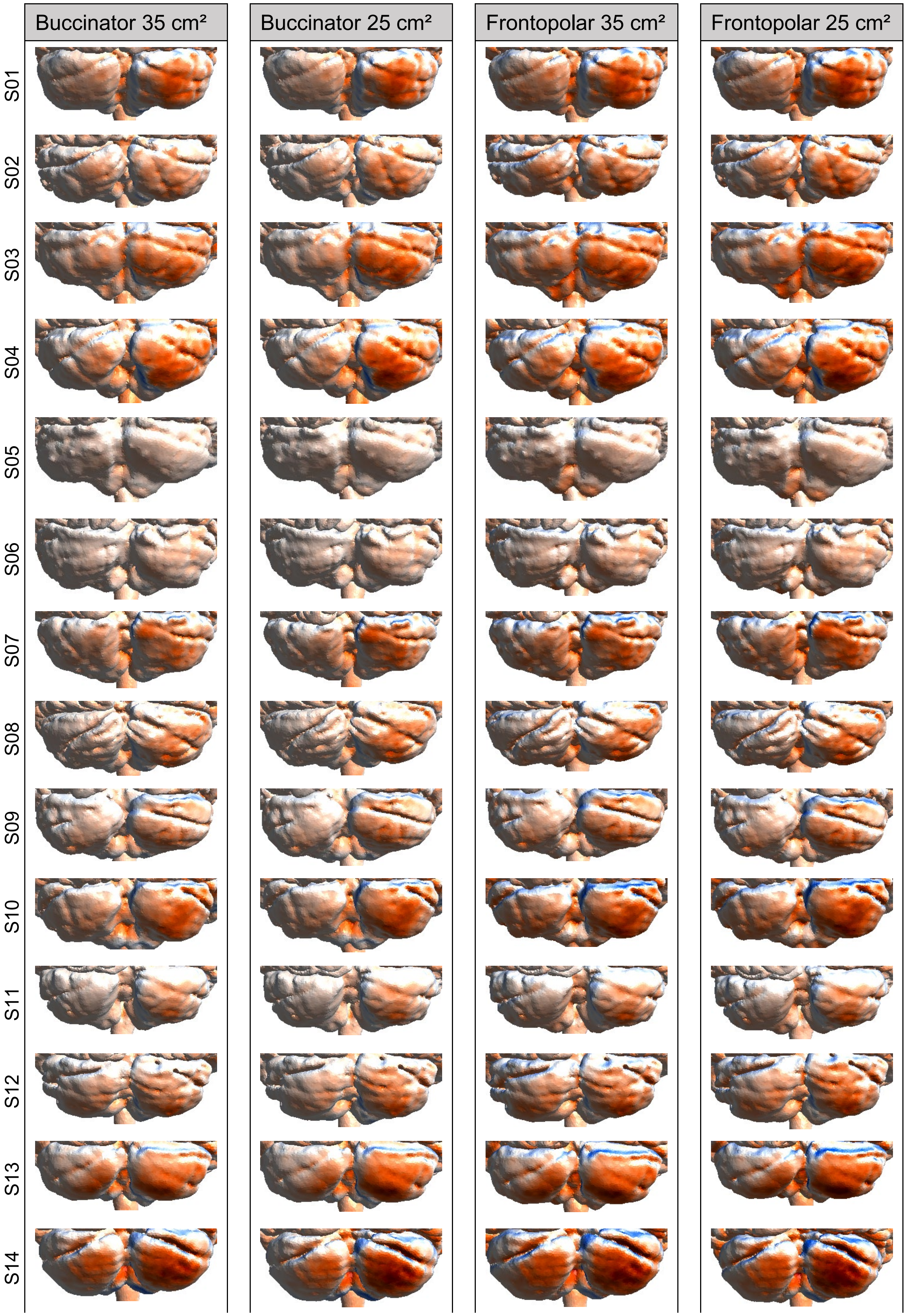

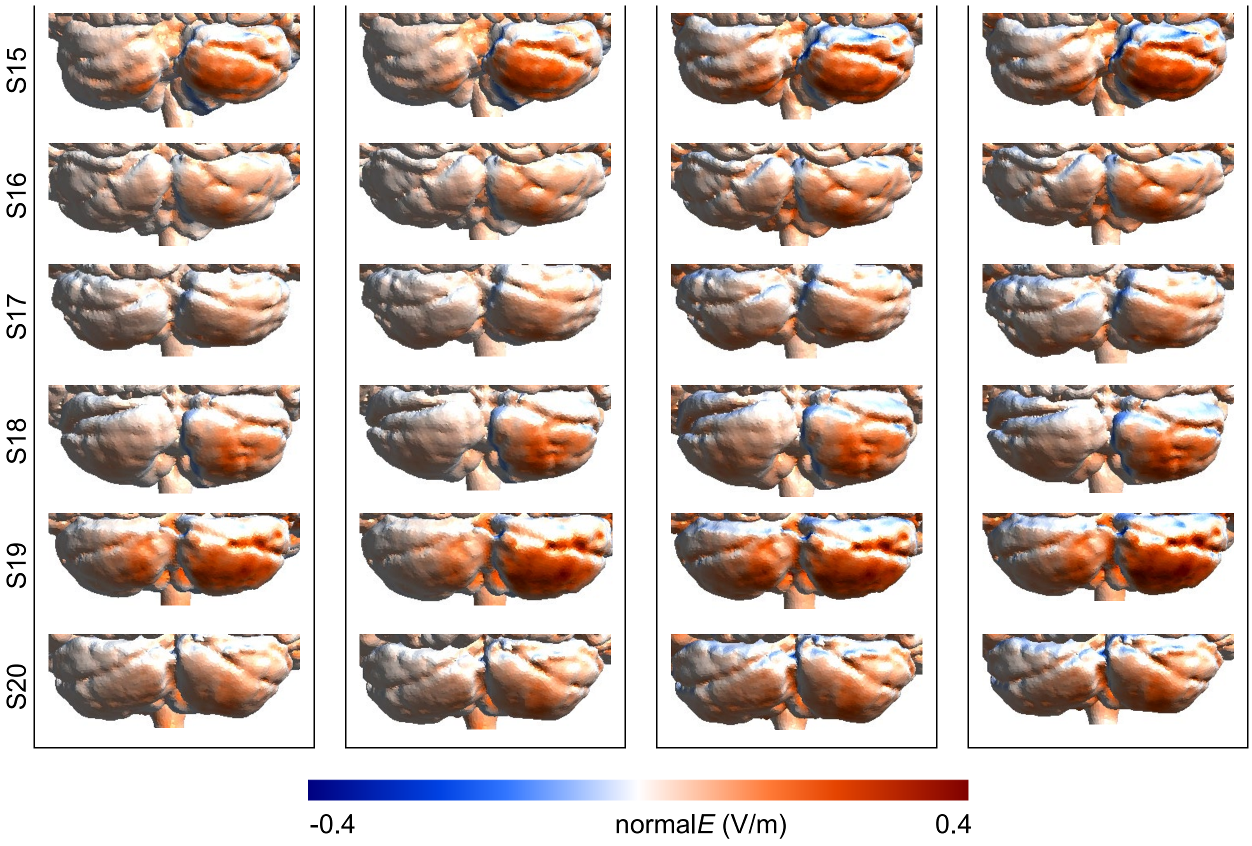
S15
S16
S17
S18
S19
S20
-0.4
normal$E$ (V/m)
0.4

Figure S 3. Electric field distributions for the six alternative montages tested in Experiment 2.

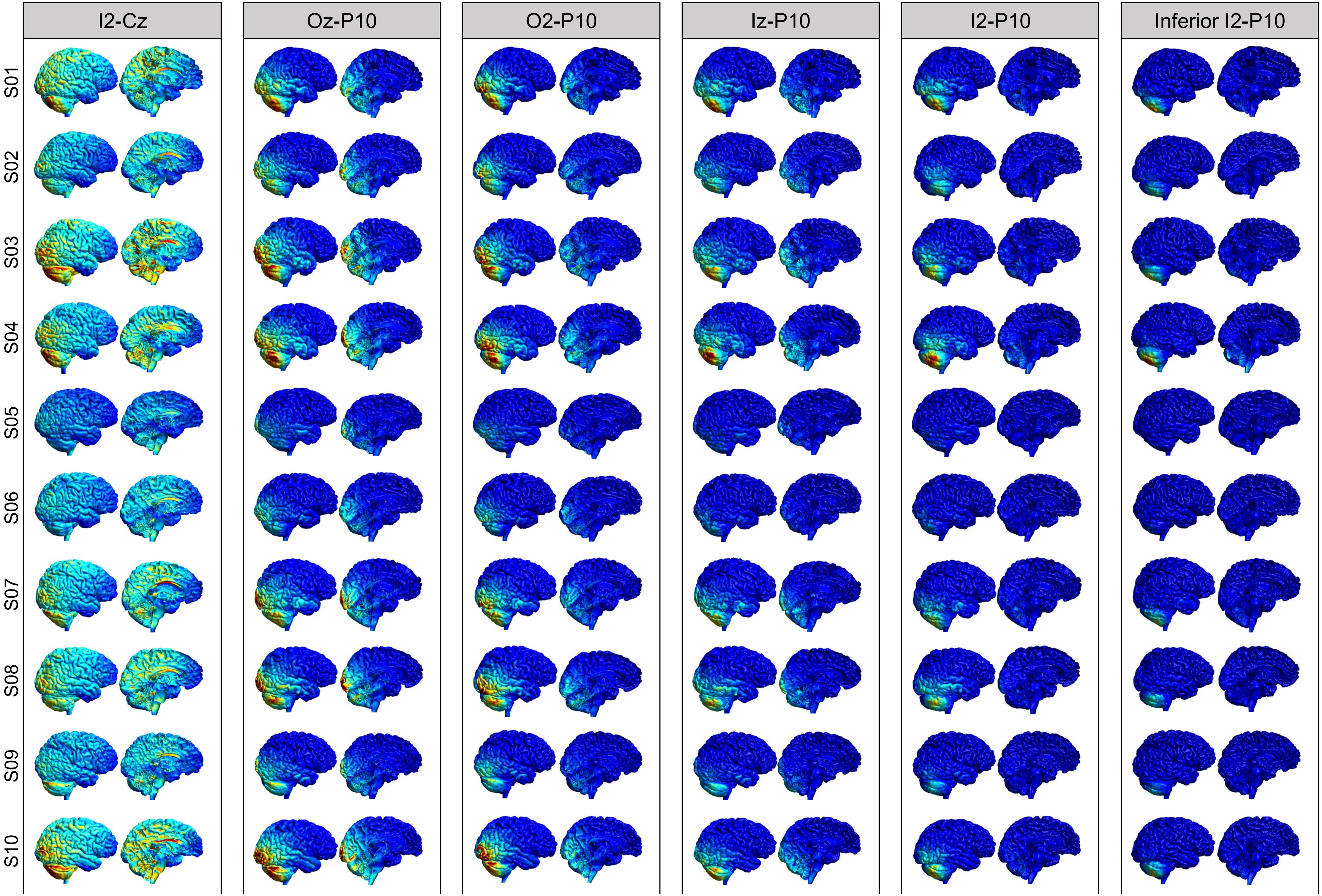

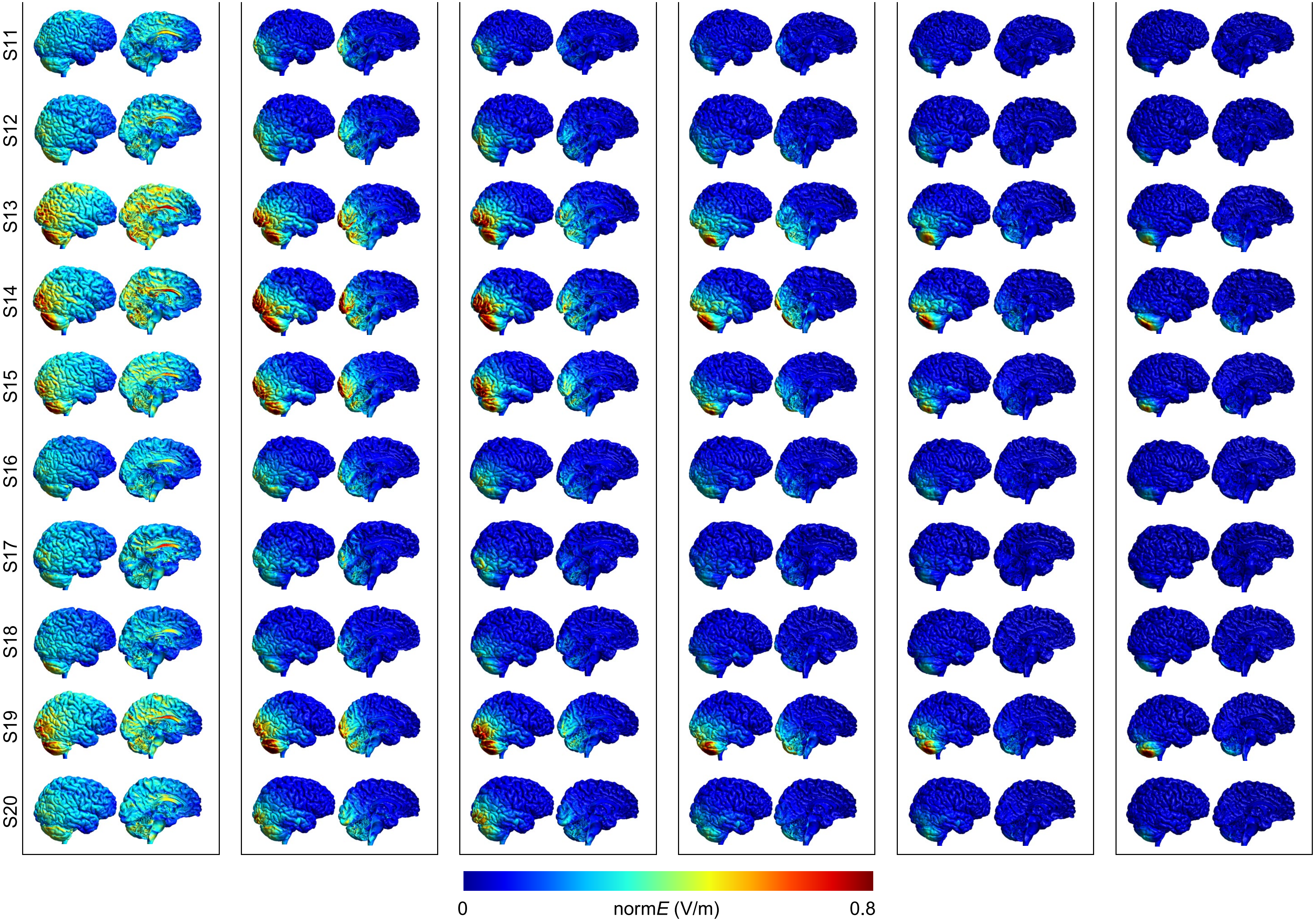
S11
S12
S13
S14
S15
S16
S17
S18
S19
S20
0
normE (V/m)
0.8

Figure S 4. Electric field directions for the six alternative montages tested in Experiment 2.

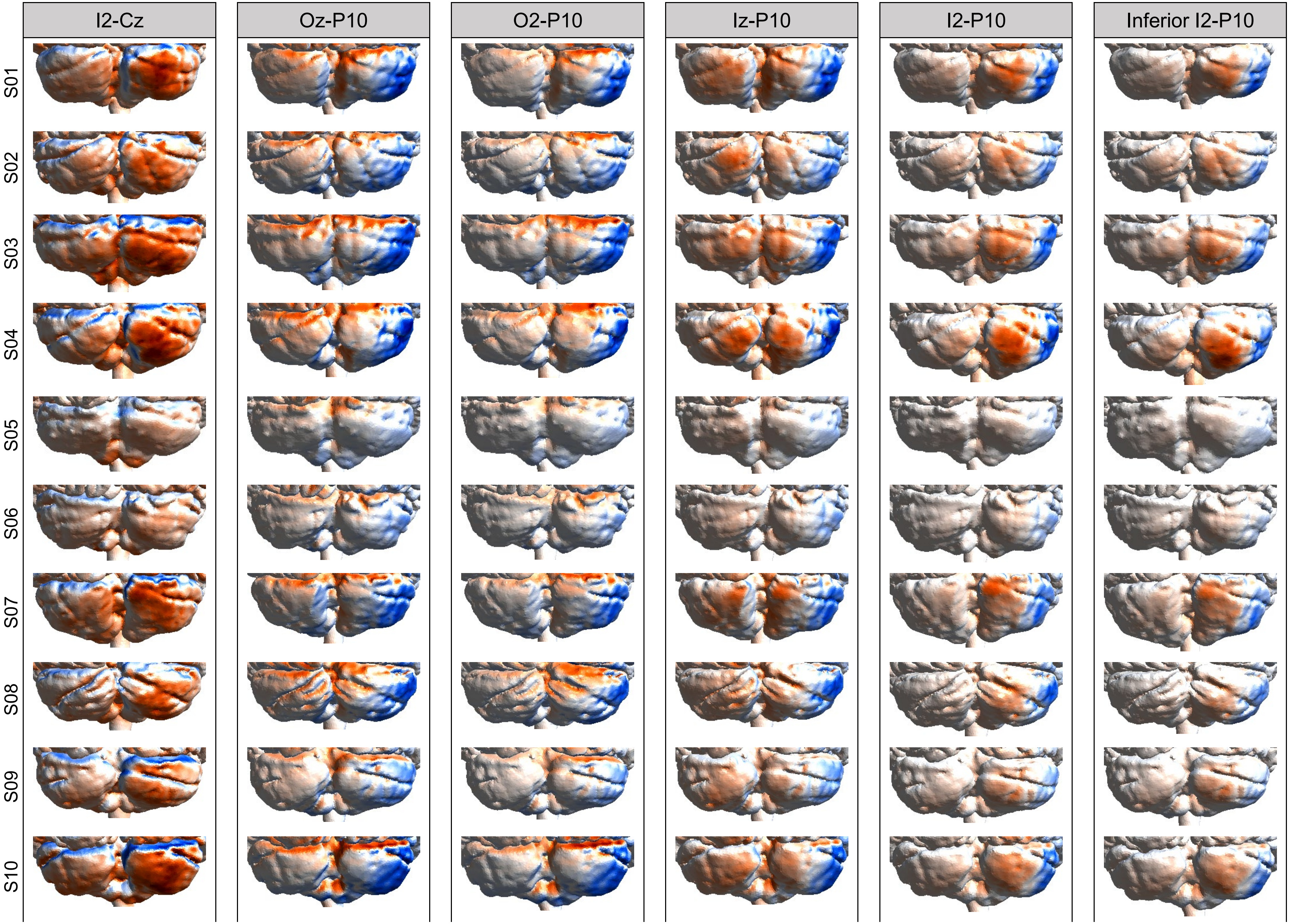

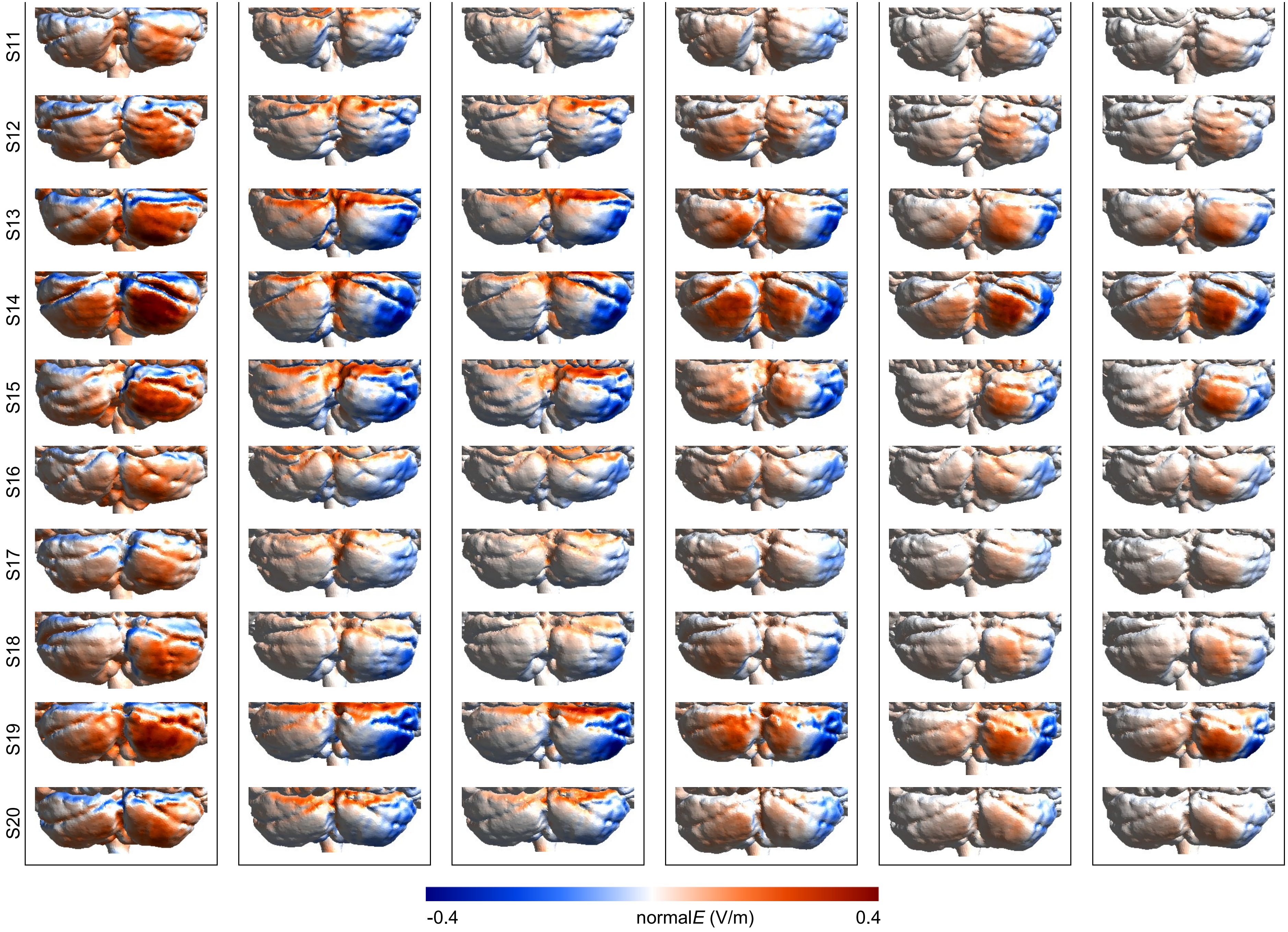
S11
S12
S13
S14
S15
S16
S17
S18
S19
S20
-0.4
normalE (V/m)
0.4

Figure S5. Field focality corresponding to the area of the grey matter with field strengths equal to or higher than 50% of the 99.9th percentile for all tested montages.

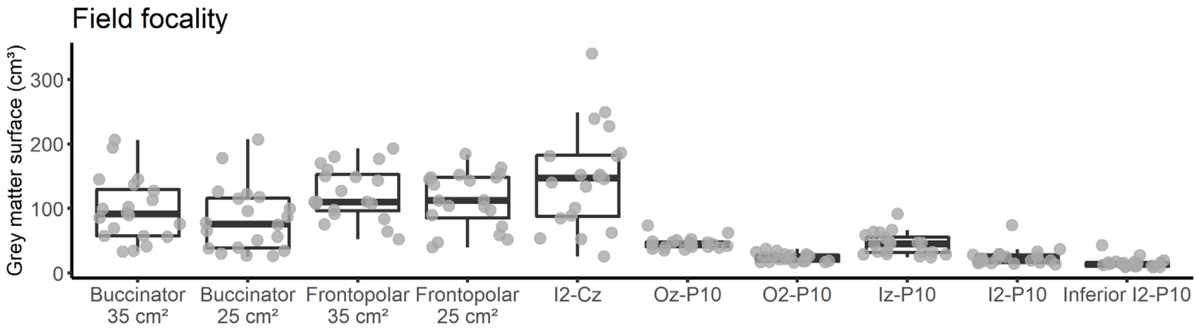

Supplement: Supplementary file 1 — Supplementary Figures. [file 41598_2021_1755_MOESM1_ESM.pdf]
